# Onset timing of letter processing in auditory and visual sensory cortices

**DOI:** 10.3389/fnint.2024.1427149

**Published:** 2024-11-14

**Authors:** Tommi Raij, Fa-Hsuan Lin, Benjamin Letham, Kaisu Lankinen, Tapsya Nayak, Thomas Witzel, Matti Hämäläinen, Jyrki Ahveninen

**Affiliations:** ^1^MGH/MIT/HMS Athinoula A. Martinos Center for Biomedical Imaging, Charlestown, MA, United States; ^2^Physical Sciences Platform, Sunnybrook Research Institute, Toronto, ON, Canada; ^3^Department of Medical Biophysics, University of Toronto, Toronto, ON, Canada; ^4^Institute of Medical Science, University of Toronto, Toronto, ON, Canada; ^5^Harvard-MIT Division of Health Sciences and Technology, Cambridge, MA, United States; ^6^Department of Neuroscience and Biomedical Engineering, Aalto University School of Science, Espoo, Finland

**Keywords:** audiovisual interaction, cross-modal, language, MEG, multisensory

## Abstract

Here, we report onset latencies for multisensory processing of letters in the primary auditory and visual sensory cortices. Healthy adults were presented with 300-ms visual and/or auditory letters (uppercase Roman alphabet and the corresponding auditory letter names in English). Magnetoencephalography (MEG) evoked response generators were extracted from the auditory and visual sensory cortices for both within-modality and cross-sensory activations; these locations were mainly consistent with functional magnetic resonance imaging (fMRI) results in the same subjects. In the primary auditory cortices (Heschl’s gyri) activity to auditory stimuli commenced at 25 ms and to visual stimuli at 65 ms (median values). In the primary visual cortex (Calcarine fissure) the activations started at 48 ms to visual and at 62 ms to auditory stimuli. This timing pattern suggests that the origins of the cross-sensory activations may be in the primary sensory cortices of the opposite modality, with conduction delays (from one sensory cortex to another) of 17–37 ms. Audiovisual interactions for letters started at 125 ms in the auditory and at 133 ms in the visual cortex (60–71 ms after inputs from both modalities converged). Multivariate pattern analysis suggested similar latency differences between the sensory cortices. Combined with our earlier findings for simpler stimuli (noise bursts and checkerboards), these results suggest that primary sensory cortices participate in early cross-modal and interaction processes similarly for different stimulus materials, but previously learned audiovisual associations and stimulus complexity may delay the start of the audiovisual interaction stage.

## 1 Introduction

Letters of the alphabet are the basic building blocks of written phonetic language. The associations between auditory and visual letters are arbitrary, language-dependent, and based on extensive learning. For these reasons, brain representations of letters have been used in studies of multisensory processing, learning, distributed representations of supramodal concepts, and dyslexia (Raij, 1999; Raij et al., 2000; van Atteveldt et al., 2004; Herdman et al., 2006; Blau et al., 2010; Andres et al., 2011; Blomert, 2011; Froyen et al., 2011).

While the traditional view was that primary sensory areas show strict sensory fidelity, i.e., they can only be activated by stimuli in the appropriate sensory modality, it is now widely accepted that even low-order sensory areas may show cross-sensory (i.e., cross-modal) activations and multisensory interactions starting as early as about 40–50 ms after stimulus onset, in both nonhuman primates (Schroeder et al., 2001; Schroeder and Foxe, 2002) and humans (Giard and Peronnet, 1999; Foxe et al., 2000; Molholm et al., 2002; Teder-Sälejärvi et al., 2002; Molholm et al., 2004; Murray et al., 2005; Talsma et al., 2007; Cappe et al., 2010; Raij et al., 2010; Beer et al., 2013). Supporting evidence comes from fMRI results showing cross-sensory activations in or very close to primary sensory areas (Pekkola et al., 2005; Martuzzi et al., 2006; Raij et al., 2010). These findings are consistent with functional and structural MRI connectivity analyses indicating direct connections between the primary auditory and visual cortices (Eckert et al., 2008; Beer et al., 2011; Beer et al., 2013). This suggest that low-order sensory areas may contribute to multisensory integration starting from very early processing stages (Schroeder et al., 2003; Foxe and Schroeder, 2005; Macaluso and Driver, 2005; Molholm and Foxe, 2005; Schroeder and Foxe, 2005; Ghazanfar and Schroeder, 2006; Macaluso, 2006; Kayser and Logothetis, 2007; Musacchia and Schroeder, 2009; Raij et al., 2010). However, the types of stimuli utilized in these studies have been limited, and it is unclear if audiovisual learning influences these processes. Consequently, the functional roles of such early multisensory activations remain elusive.

Determining onset latencies of stimulus-evoked responses is a robust way of detecting the order and spread of activations across different brain areas. We have previously reported onset latencies for audiovisual processing of noise bursts and checkerboard stimuli (Raij et al., 2010). However, such simple stimuli, while strongly activating sensory systems, do not have learned audiovisual associations, which may influence multisensory interactions (Raij et al., 2000; van Atteveldt et al., 2004). Letters are physically relatively simple and therefore allow meaningful comparisons with studies utilizing simpler stimuli; yet, they have strong audiovisual associations formed through extensive learning. Therefore, we here examine onset latencies for letters of the alphabet. To improve comparability between studies, the subjects, experimental design, and recordings are identical to our earlier experiment with simpler stimuli (Raij et al., 2010), together with the present data forming a multimodal MRI/fMRI/MEG dataset on multisensory processing. The present data were recorded in the same session as in Raij et al. (2010) and were previously used in a different study (Lankinen et al., 2024).

## 2 Materials and methods

### 2.1 Subjects, stimuli, and tasks

The protocol was approved by the Massachusetts General Hospital institutional review board, and subjects gave their written informed consent prior to participation. The visual stimuli were individual uppercase letters of the Roman alphabet (visual angle 3.5°× 3.5°, contrast 100%, foveal presentation) and auditory stimuli the corresponding spoken English letter names. Sixteen different letters were used (ADEFIKLMNORSTVXY). The auditory stimuli were recorded from a female speaker in an echoless chamber at the Department of Cognitive and Neural Systems at Boston University. The duration of all stimuli was 300 ms. Auditory stimulus onset was defined as the onset of the first audible component for each letter; as expected, the envelope profiles following onsets varied across auditory letters. The letters were presented as auditory only (A), visual only (V), or audiovisual combination (AV, simultaneous auditory and visual, always congruent), in a rapid event-related fMRI-type design with pseudorandom stimulus order and interstimulus interval (ISI). A/V/AV stimuli were equiprobable. Subjects were 8 healthy right-handed humans (6 females, age 22–30). The task was to respond to rare (10%) auditory (sound [kei]), visual (letter K), or audiovisual ([kei]/K) target stimuli (A Target / V Target / AV Target) with the right index finger as quickly as possible while reaction time (RT) was measured. All subjects were recorded with three stimulus sequences having different interstimulus intervals (ISIs) with mean ISIs at 1.5/3.1/6.1 s, across which the MEG onset latencies were practically identical and were therefore averaged within subjects. Inside each sequence the ISI was jittered at 1.15 s (1 TR of the fMRI acquisition) to improve fMRI analysis power (Dale, 1999; Burock and Dale, 2000). Identical stimuli and tasks were used in MEG and fMRI. Visual stimuli were projected with a video projector on a translucent screen. The auditory stimuli were presented with MEG-compatible headphones or through MRI-compatible headphones (MR Confon GmbH, Magdeburg, Germany). Auditory stimuli were adjusted to be as loud as the subject could comfortably listen to (in MEG about 65 dB SPL; in fMRI clearly above the scanner acoustical noise). Stimuli were presented with a PC running Presentation 9.20 (Neurobehavioral Systems Inc., Albany, CA, USA.). In fMRI stimuli were synchronized with triggers from the fMRI scanner. Timing of the stimuli with respect to trigger signals was confirmed with a digital oscilloscope. To maximize comparability, all parameters (except the stimuli) were identical to those used in our previous study utilizing simpler stimuli (Raij et al., 2010).

### 2.2 Structural MRI recordings and analysis

Structural T1-weighted MRIs were acquired with a 1.5 T Siemens Avanto scanner (Siemens Medical Solutions, Erlangen, Germany) and a head coil using a standard MPRAGE sequence. Anatomical images were segmented with the FreeSurfer software (Fischl et al., 2002; Fischl et al., 2004). Individual brains were spatially co-registered by morphing them into the FreeSurfer average brain via a spherical surface (Fischl et al., 1999).

### 2.3 fMRI recordings and analysis

Brain activity was measured using a 3.0 T Siemens Trio scanner with a Siemens head coil and an echo planar imaging (EPI) blood oxygenation level dependent (BOLD) sequence (flip angle 90°, TR = 1.15 s, TE = 30 ms, 25 horizontal 4-mm slices with 0.4 mm gap, 3.1×3.1 mm in-plane resolution, fat saturation off). fMRI data were analyzed with FreeSurfer. During preprocessing, data were motion corrected (Cox and Jesmanowicz, 1999), spatially smoothed with a Gaussian kernel of full-width at half maximum (FWHM) 5 mm, and normalized by scaling the whole brain intensity to a fixed value of 1,000. The first three images of each run were excluded as were (rare) images showing abrupt changes in intensity. Any remaining head motion was used as an external regressor. A finite impulse response (FIR) model (Burock and Dale, 2000) was applied to estimate the activations as a function of time separately for each trial type (A /V/AV/A Target/V Target/AV Target) with a time window of 2.3 s pre-stimulus to 16.1 s post-stimulus. The functional volumes were spatially aligned with the structural MRI of individual subjects. During group analysis, the individual results were morphed through a spherical surface into the FreeSurfer average brain (Fischl et al., 1999) and spatially smoothed at 10 mm FWHM. To enhance comparability, these parameters were identical to those used in our previous study (Raij et al., 2010).

### 2.4 MEG recordings and alignment with MRI

The MEG equipment, recordings, and analyses were the same as those reported previously (Raij et al., 2010). Whole-head 306-channel MEG (VectorView, Elekta-Neuromag, Finland) was recorded in a magnetically shielded room (Cohen et al., 2002; Hämäläinen and Hari, 2002). The instrument employs three sensors (one magnetometer and two planar gradiometers) at each of the 102 measurement locations. We also recorded simultaneous horizontal and vertical electro-oculogram (EOG). All signals were band-pass filtered to 0.03–200 Hz prior to sampling at 600 Hz. Prior to the MEG recordings, the locations of four small head position indicator (HPI) coils attached to the scalp and several additional scalp surface points were recorded with respect to the fiduciary landmarks (nasion and two preauricular points) using a 3-D digitizer (Fastrak Polhemus, VT). For MRI/MEG coordinate system alignment, the fiduciary points were then identified from the structural MRIs. Using scalp surface locations, this initial approximation was refined using an iterative closest point search algorithm.

### 2.5 MEG evoked response analysis (sensor space)

The MEG responses were averaged offline separately for each trial type (A/V/AV/A Target/V Target/AV Target), time locked to the stimulus onsets, from 250 ms pre-stimulus to 1,150 ms post-stimulus. A total of 375 individual trials per category were recorded for the non-target conditions (100 epochs for the long, 125 for the intermediate, and 150 for the short ISI run). Trials exceeding 150 μV or 3,000 fT/cm at any EOG or MEG channel, respectively, were automatically discarded. The averaged signals were digitally low-pass filtered at 40 Hz and amplitudes measured with respect to a 200-ms pre-stimulus baseline. Onset timings were analyzed separately for the responses to A, V, and AV stimuli. Specifically, for the sensor space analysis, we estimated onsets from the amplitudes $\surd \left(b_{x^{2}}+b_{y^{2}}\right)$ computed from the signals *b_x_* and *b_y_* of the two planar gradiometers at each sensor location (Supplementary material for details). From each subject, 3 sensor locations were selected showing maximal responses over the primary auditory cortices (1 location in each hemisphere) and the primary visual cortex (1 location at posterior midline). Onset latencies were picked at the first time point that exceeded three standard deviations (3SDs) above noise level estimated from the 200-ms pre-stimulus baseline. In addition, we required that the response must not start earlier than 15 ms (based on finite conduction delays in sensory pathways) and the response has to stay above the noise level for at least 20 ms (to protect against brief noise spikes). The onset latency analyses were done separately for the grand average time course (averaged across all accepted conditions and subjects to improve the signal-to-noise ratio SNR) and for the individual level responses (to allow computation of variability across subjects but with lower SNR). Data from one subject were too noisy for accurate onset latency determination and were therefore discarded; the same subject was discarded in the previous publication utilizing simpler stimuli. Additionally, in two subjects, one run (out of the total of three runs with different ISIs) was contaminated by eye blinks and therefore discarded; in these subjects only the data from the remaining two runs were used. After combining the runs with different ISIs, each subject’s averaged response consisted of about 300 trials (for details see Supplementary material).

Finally, to estimate AV interactions, we calculated the AV interaction responses $\left[AV-\left(A+V\right)\right]$ from their constituent A, V, and AV evoked responses. Such responses have been used widely to study multisensory interactions since their introduction (Morrell, 1968). However, addition and subtraction of responses decreases signal-to-noise ratios (for interaction responses, theoretically by √3 times over their constituent A, V and AV responses). Therefore, the AV interaction responses were filtered more strictly (low-pass filtering at 20 Hz with 3 dB roll-off) and were excluded from sensor space analysis for onset latencies; instead, the sensor space AV interaction responses were subjected to source analysis (see below) followed by extraction of their onsets.

### 2.6 MEG source analysis and onset latencies (source space)

As in our earlier study (Raij et al., 2010), minimum-norm estimates (MNEs) (Hämäläinen and Ilmoniemi, 1984; Hämäläinen and Ilmoniemi, 1994) were computed from combined anatomical MRI and MEG data (Dale and Sereno, 1993; Liu et al., 1998; Dale et al., 2000) using the MNE software (Gramfort et al., 2014). The noise normalized MNE (dSPM) values were then calculated to reduce the point-spread function and to allow displaying the activations as an *F*-statistic. The individual dSPM results were morphed through a spherical surface into the FreeSurfer average brain (Fischl et al., 1999). Grand average dSPM estimates were calculated from the grand average MNE and the grand average noise covariance matrix. The dSPM time courses were calculated separately for A, V, AV, and AV interaction responses and extracted from anatomically pre-determined (Desikan et al., 2006) locations of A1 and V1. Next, their onset latencies were measured as described above for sensor signals using the 3SD and latency minimum criteria. These values were extracted separately from grand average time courses (averaged across subjects) and from the individual level data. Finally, to extract the onset latencies from the source-space signals with high SNR while also being able to estimate their variances, we additionally used bootstrapping (for details see Supplementary material).

### 2.7 MVPA analysis of MEG source space data

We also examined early cross-sensory influences using multivariate pattern analysis (MVPA). MVPA decoding analysis was performed by two-class support vector machine (SVM) classifier with linear kernel and cost equal to one (C = 1) implemented in libsvm (Chang and Lin, 2011) and provided in the CoSMoMVPA package^1^ (Oosterhof et al., 2016) in MATLAB (Natick, MA, USA). The decoding was performed separately for each subject, with bilateral A1:s and V1:s as the ROIs. The individual-trial MEG source estimates were averaged within each hemisphere-specific ROI before MVPAs. For this analysis, only the two runs with longer ISIs were selected, because the recordings with the shortest ISI would have had crosstalk between trials. The classification was conducted using a temporal searchlight analysis with a sliding window width of 50 ms with the window moved at 1.7 ms steps (the sampling rate). For contrasts that required a “noise” sample, the noise data were drawn from the time segments between stimuli. In each of our 100 randomized cross-validation folds, the model was trained in 80% of the trials and tested in the remaining 20% of trials. The decoding accuracy was averaged across the 100 folds. We tested the statistical significance for each subject using a *t-*test with a chance level of 0.5. Finally, the *p-*values were corrected for multiple comparisons using the false discovery rate (FDR) procedure (Benjamini and Hochberg, 1995).

## 3 Results

### 3.1 Behavioral results

The average hit rate to target stimuli was 97% across all experimental conditions. During MEG, the RTs were faster for AV (median 487 ms, mean ± SD 492 ± 65 ms) than for A (median 576 ms, mean ± SD 579 ± 99 ms) and V (median 518 ms, mean ± SD 530 ± 73 ms) stimuli with outliers excluded according to the Median Absolute Deviation (MAD) statistics criterion. In fMRI the difference was slightly smaller (for AV median 599 ms, mean 603 ± 80 ms, for A median 700 ms, mean 722 ± 128 ms, and for V stimuli median 615 ms, mean 623 ± 86 ms). Similar as in our earlier study (Raij et al., 2010), the longer RTs in fMRI may be due to slower response pads and the MR environment. Since the A, V, and AV stimuli were in random order and stimulus timing was pseudorandom, attention related or anticipatory differences could not have influenced the onset latency differences across conditions.

### 3.2 MEG onset latencies: sensor data

**Figure 1:**
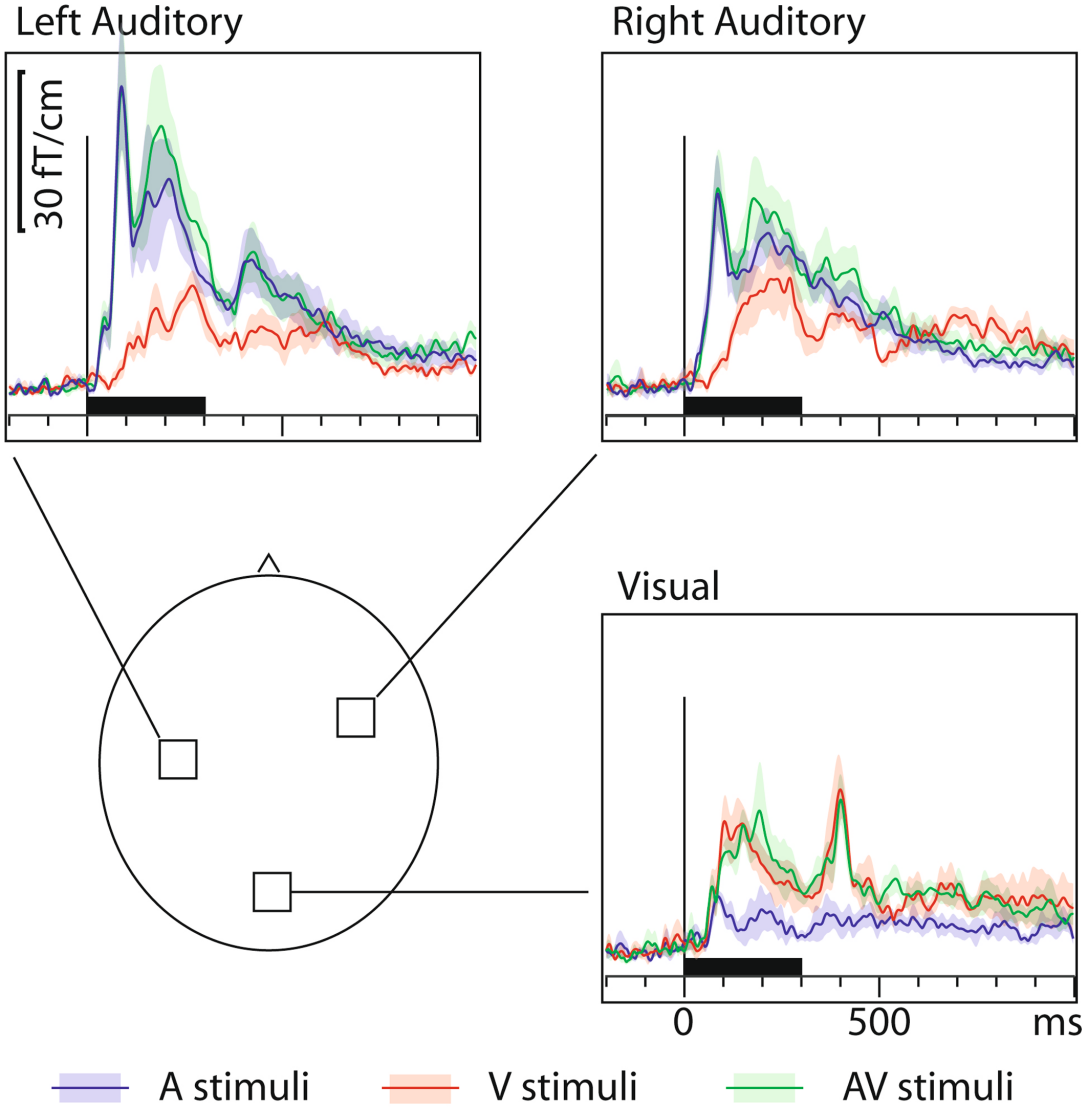
MEG grand average sensor response (gradient amplitude) time courses with across-subjects means (lines) and their standard error of the mean (SEM) error bars (shaded areas around the mean curves) over the auditory and visual cortices for auditory (blue traces), visual (red traces), and audiovisual (green traces) letters. The approximate sensor locations are shown in the lower left panel. The corresponding onset latency numerical values are listed in Table 1. To generate these grand average waveforms (*N*=7), from each subject, the sensor location showing the maximal ~100 ms sensory-specific response was selected, and the signals from these sensors were averaged across subjects. Sensors over both auditory and visual cortices show cross-sensory activations, but these are stronger over the auditory than the visual cortex. The sensory-specific activations occur earlier than the cross-sensory activations. Time scales –200 to +1000 ms post stimulus, stimulus duration 300 ms (black bar).

Figure 1 shows that, in addition to the expected sensory-specific activations, cross-sensory effects were observed: visual stimuli strongly activated temporal cortices and auditory stimuli (albeit more weakly) the midline occipital cortex. Table 1 lists the corresponding onset latencies (the time when the grand average response first exceeded 3SD above the noise level). The sensory-specific activations started 34 ms earlier over the auditory than visual cortex. The cross-sensory activations started after the sensory-specific responses, by 3 ms over visual cortex and by 49 ms over auditory cortex. Table 2 lists the across-subjects onset latencies; the left and right auditory cortices showed similar timings and were thus averaged. The individual subjects’ sensor-level responses were relatively noisy and thus did not correspond well to the grand average results; hence, no statistical comparisons were done for the sensor data (see dSPM data below for statistical tests across individuals).

**Table 1:** Sensor onset latencies (ms) over the auditory and visual cortices.

Sensor latencies (ms) Grand average responses	Auditory cortex		Visual cortex
	Left	Right	
Auditory stimuli	27	25	63
Visual stimuli	80	70	60
AV stimuli	27	30	55

The values were picked from the grand average time courses shown in Figure 1; for individual subject values, see Table 2.

**Table 2:** Across-subjects sensor onset latencies (ms) over the auditory and visual cortices.

Sensor latencies (ms) Across-subjects values	Auditory cortex Mean ± SD (median)	Visual cortex Mean ± SD (median)
Auditory stimuli	38 ± 17 (32)	76 ± 39 (65)
Visual stimuli	91 ± 11 (93)	63 ± 10 (65)
AV stimuli	39 ± 15 (33)	66 ± 31 (57)

These values were picked from the individual level responses.

### 3.3 MEG dSPM source analysis

**Figure 2:**
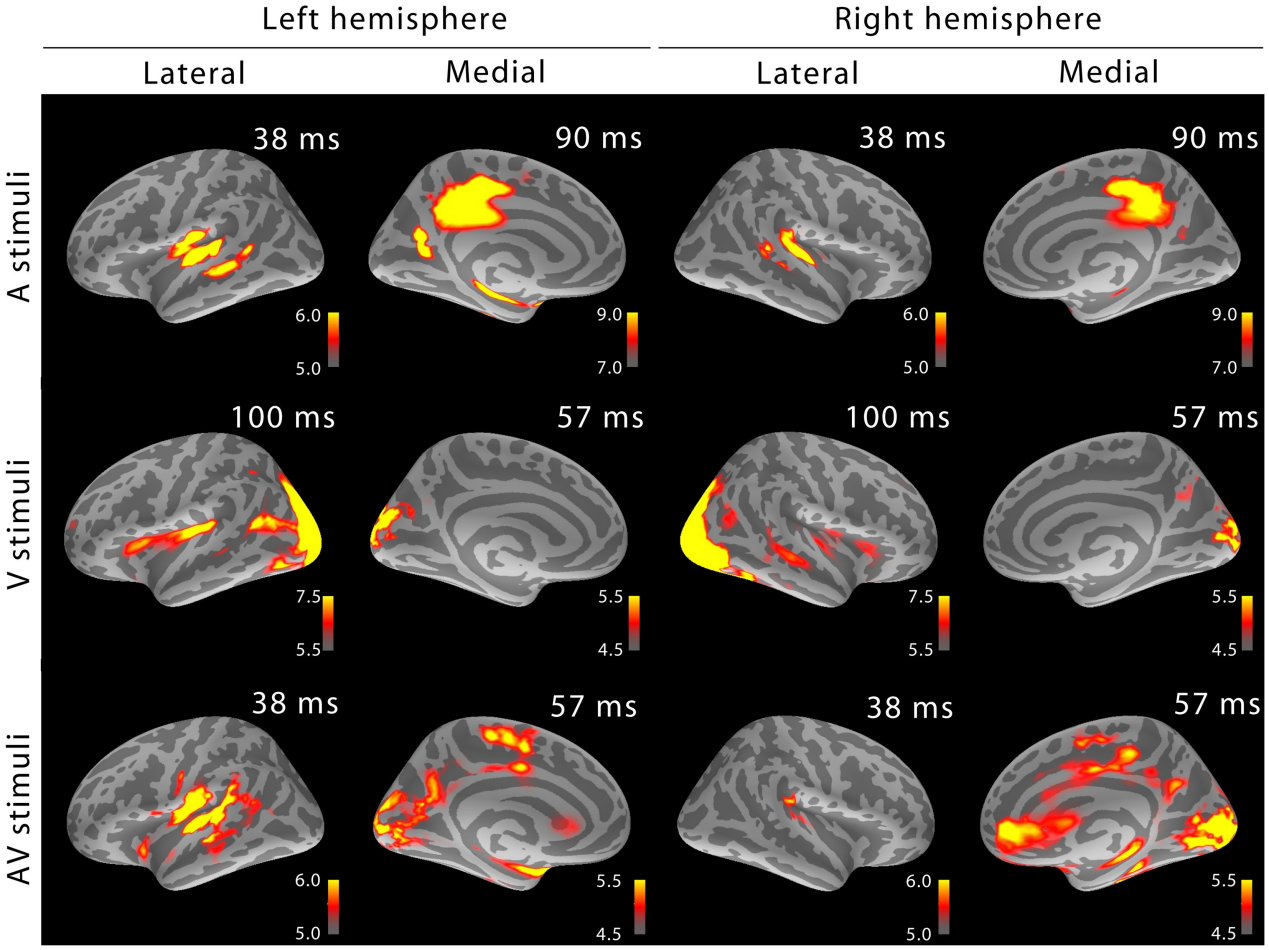
MEG snapshots (dSPM √F-statistics) at early activation latencies. Both sensory-specific and cross-sensory activations are seen at primary sensory areas (the right calcarine cortex cross-sensory activity is not visible at this threshold). While some of the cross-sensory activations are located inside the sensory areas (as delineated in Desikan et al., 2006), other activations occupy somewhat different locations than the sensory-specific activations. However, the spatial resolution of MEG is somewhat limited – hence exact comparisons are discouraged. Outside sensory areas, the cross-sensory activations to visual stimuli show additional bilateral activations in superior temporal sulci (STS, more posterior in the left hemisphere) and left Broca’s area. Grand average data (*N*=7).

Figure 2 shows the MEG localization results at selected time points after the onset of activity. Similar as for sensor space analysis, in addition to sensory-specific activations, cross-sensory activations were observed: visual stimuli strongly activated large areas of temporal cortex including the supratemporal auditory cortex, and auditory stimuli, albeit more weakly, some parts of the calcarine fissure especially in the left hemisphere (right hemisphere cross-sensory activity in calcarine cortex was below selected visualization threshold). Additional cross-sensory activations were observed outside the primary sensory areas.

### 3.4 MEG dSPM source-specific onset latencies

**Figure 3:**
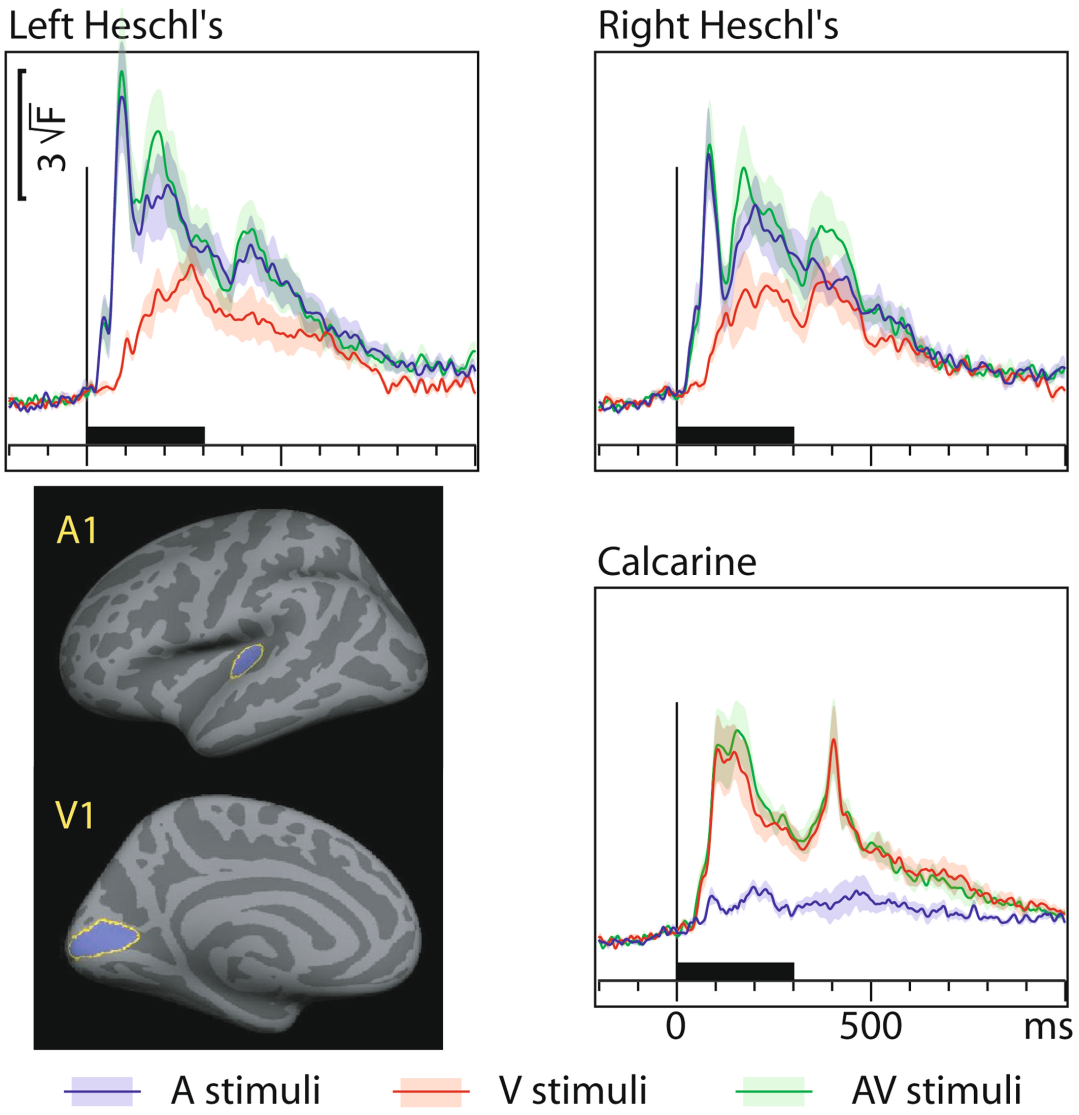
MEG grand average source-specific dSPM time courses and their across-subjects SEM error bars for Heschl’s gyri (A1) and calcarine fissure (V1) to auditory, visual, and audiovisual stimuli. The source areas, shown for the left hemisphere in the lower left panel, were based on an anatomical parcellation (Desikan et al., 2006); left and right calcarine sources were averaged. The corresponding onset latency numerical values are listed in Table 3. Both sensory-specific and cross-sensory activations are observed. The sensory-specific activations occur earlier than the cross-sensory activations. Time scales –200 to +1000 ms post stimulus, stimulus duration 300 ms (black bar). Grand average data (*N*=7) showing means (lines) and SEM error bars (shaded areas around the mean time courses).

Figure 3 shows MEG dSPM time courses from Heschl’s gyri (HG/A1) and calcarine fissure (V1) for auditory, visual, and AV stimuli. The left and right calcarine fissure activations, due to their close anatomical proximity and similar timings, were averaged. As expected, the time courses are similar to the sensor amplitudes in Figure 1. However, in the presence of multiple sources, dSPM time courses more accurately allow extraction of activity from a specific area than sensors that collect activity from a rather large area. Table 3 lists the corresponding onset latencies measured from the grand average dSPM responses using bootstrapping. Since onsets were very similar across hemispheres, the responses were averaged across the left and right hemisphere. Sensory-specific activations started 23 ms (median) earlier in A1 than in V1. In V1, sensory-specific activations started 14 ms before the cross-specific responses, whereas in A1 cross-sensory activations started 40 ms after the sensory-specific responses. Cross-modal conduction delays (spread from one sensory cortex to another) were 37 ms for auditory and 17 ms for visual stimuli. As expected, onsets of responses to AV stimuli closely followed onsets to the unimodal stimulus that first reached the sensory cortex.

**Table 3:** Across-subjects onset latency means ± SDs (Medians) in source space.

Source latencies (ms) Across-subjects values using bootstrapping	Heschl’s gyrus Mean ± SD (Median)	Calcarine cortex Mean ± SD (Median)
Auditory stimuli	25 ± 2 (25)	55 ± 18 (62)
Visual stimuli	58 ± 17 (65)	48 ± 6 (48)
AV stimuli	25 ± 2 (25)	46 ± 4 (45)
AV Interaction (20 Hz LPF)	131 ± 27 (125)	126 ± 56 (133)

In this analysis the evoked responses from each hemisphere and subject were bootstrapped. In addition, AV interaction responses were low-pass filtered at 20 Hz to mitigate SNR problems caused by the $\left[AV-\left(A+V\right)\right]$ operation. For the corresponding values for A/V/AV stimuli extracted from the individual subject values without bootstrapping, see Table 4.

**Figure 4:**
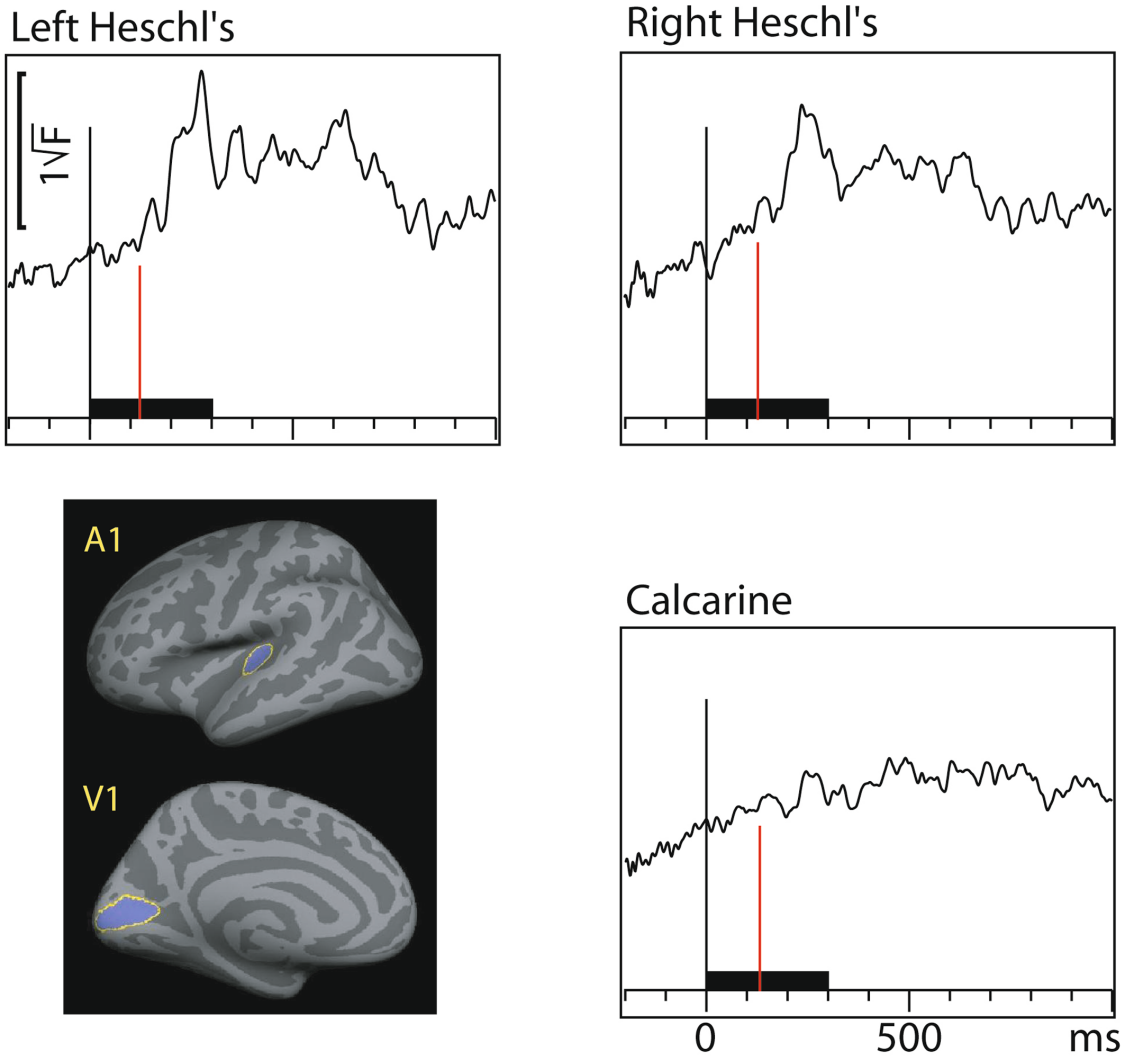
Audiovisual interaction [AV– (A+V)] time courses (MEG source-specific dSPM) from Heschl’s gyri (A1) and calcarine fissure (V1); the onset latencies are reported in Table 3 (with bootstrapping, left and right A1 averaged). Interactions are observed in both the auditory and visual cortices. Time scales –200 to +1000 ms post stimulus, stimulus duration 300 ms (black bar). Grand average data (*N*=7).

Figure 4 shows dSPM time courses calculated from the audiovisual interaction responses. As expected, these were weaker than the constituent A/V/AV responses. In the auditory cortex the interactions started 60 ms after and in the visual cortex 71 ms after inputs from both sensory modalities converged.

Table 4 lists dSPM onset latencies (mean ± SD and median) across the individual subjects (without bootstrapping). On the one hand, since these values were picked from the individual subject responses where SNR is lower than in group-level averaged responses, the onsets are somewhat longer than in Table 3. On the other hand, the individual level values allow for straightforward statistical testing. Sensory-specific auditory evoked responses in A1 started 28 ms earlier than visual evoked responses in V1 (Wilcoxon signed rank test for medians (*n* = 7), *p* = 0.018). Cross-sensory activations in A1 occurred 52 ms later than sensory-specific activations, which was statistically significant (*p* = 0.028). In V1, cross-sensory activations occurred 10 ms later than sensory-specific activations, but this difference was not quite significant (*p* = 0.063). Difference between cross-modal conduction delays (from one sensory cortex to another) for auditory and visual stimuli was non-significant (*p* = 0.128).

**Table 4:** Across-subjects source-specific onset latencies (ms).

dSPM latencies (ms) Across-subjects values	Heschl’s gyrus Mean ± SD (Median)	Calcarine cortex Mean ± SD (Median)
Auditory stimuli	32 ± 13 (30)	94 ± 52 (68)
Visual stimuli	82 ± 16 (82)	56 ± 9 (58)
AV stimuli	32 ± 12 (28)	51 ± 13 (55)

These values were picked from the individual level responses.

### 3.5 MVPA results

**Figure 5:**
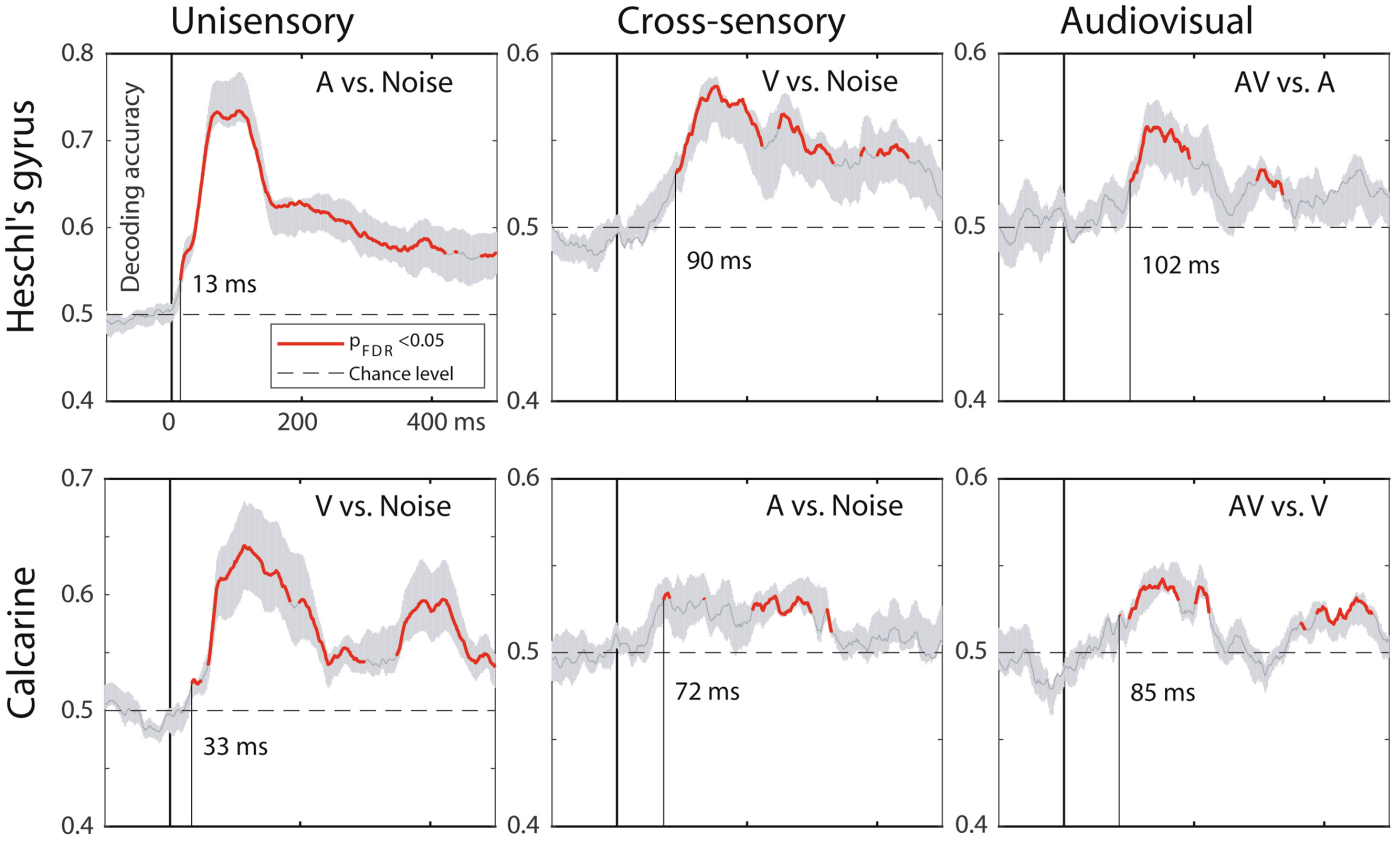
MVPA results, MEG source-space analysis in A1 (top) and V1 (bottom). The traces depict decoding accuracy over time (red = accuracy exceeds the statistical significance threshold; 0 ms = stimulus onset; post-stimulus vertical line = time when the decoding accuracy first becomes significant; grey shade = SEM across subjects). Decoding for unimodal stimuli starts in sensory-specific cortices (left column) and emerges faster in A1 than in V1. Cross-sensory decoding for unimodal stimuli (middle column) occurs later, followed by audiovisual interactions for bimodal stimuli (right column). Time scale –100 to +500 ms post stimulus, stimulus duration 300 ms, analysis time window length 50 ms, time window sliding step 1.7 ms.

Figure 5 shows the MVPA decoding results for relevant contrasts in A1 and V1. The results suggest similar processing stage and inter-regional latency differences between A1 and V1 as the ERP onsets.

### 3.6 fMRI activations

**Figure 6:**
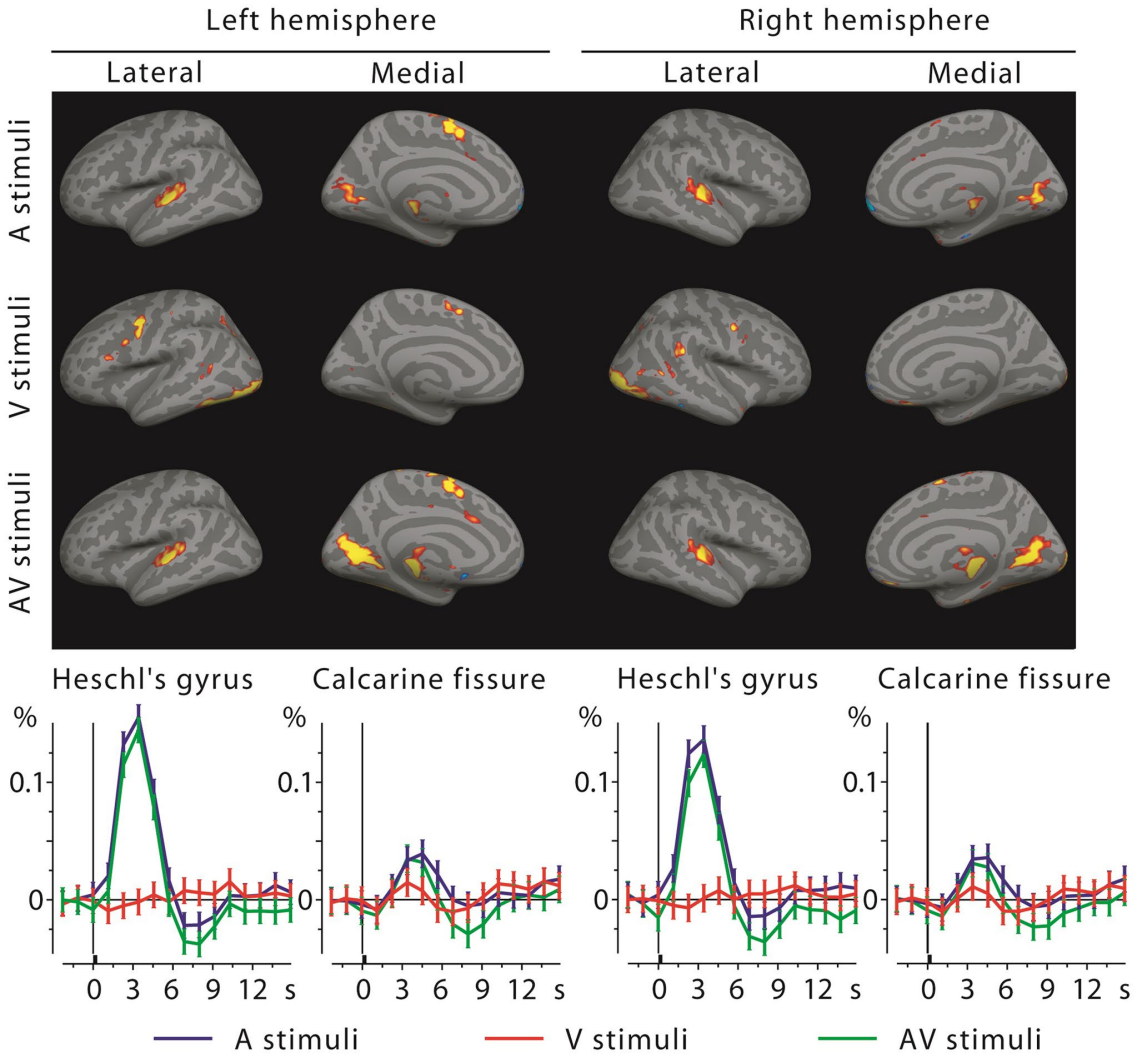
fMRI activations to auditory, visual, and audiovisual stimuli at the 4th time frame after stimulus onset (top, showing mainly positive activations at this frame) and the corresponding BOLD signal time courses with across-subjects SEM error bars from Heschl’s gyri and calcarine fissures (bottom). Sensory-specific activations are strong for auditory letters but quite weak for visual letters (in V1, below visualization threshold in brain activation maps); cross-sensory responses are clear in Calcarine fissures bilaterally, but absent in the left and weak in the right Heschl’s gyrus (see Discussion).

MEG source analysis can be complicated by the electromagnetic inverse problem and volume conduction. We therefore recorded fMRI activations using the same subjects and stimuli. Figure 6 shows the grand average fMRI results, with the BOLD time courses in the lower panels. Calcarine cortex was activated by both visual and auditory letters, and in fact more strongly by the latter. Heschl’s gyri showed strong activity for auditory stimuli, but, in contrast to the MEG results, for visual stimuli only the right hemisphere showed a small deflection at the typical BOLD signal peak latency. At closer inspection the right A1 voxels that were activated by visual letters were concentrated in the posteromedial part of Heschl’s gyrus, spatially diluting this effect when averaged across the entire ROI.

### 3.7 Comparisons for activations between letters and simpler stimuli

We have previously reported onset latencies for simpler stimuli (checkerboards and noise bursts) in the same subjects using identical paradigms and recordings as in the present study (Raij et al., 2010). The values for sensory-specific and cross-sensory activations were similar regardless of stimulus type, as can be seen comparing the values between (Raij et al., 2010) and the present study; differences were non-significant, with the exception of V1 responses to visual stimuli starting later (by 13 ms for median, 8 ms for mean) for letters than for checkerboard stimuli (Wilcoxon Signed Rank Test for medians *p* = 0.034). This difference can probably be attributed to that letters cover a smaller proportion of the visual field than checkerboards, therefore activating fewer V1 neurons and weakening the population response.

The largest latency difference was that the AV interactions started later for letters (present study) than for simpler stimuli (Raij et al., 2010). In A1 this difference was 40 ms and in V1 56 ms (bootstrapped medians). These differences were statistically significant (*p* < 0.001 separately for both A1 and V1 for the bootstrapped medians; for details of the employed statistical test see Supplementary material).

Some source distribution/amplitude differences were also evident when comparing Figures 2 between the two studies. For sensory-specific activations, in Heschl’s gyri the auditory letters evoked stronger responses than noise bursts, whereas in the Calcarine fissure, checkerboards resulted in stronger activation than visual letters. Source distributions outside primary sensory areas at early MEG latencies (Figure 2) were also somewhat different between basic stimuli and letters. Specifically, visual letters evoked a more left-lateralized response in Broca’s area and stronger responses in left posterior superior temporal sulcus (STS) than checkerboards.

In fMRI, results were also similar across the two stimulus types, but some differences were found in the activation strengths. In V1, visual evoked activations were clearly stronger for checkerboards than for letters. This may reflect that the different letters activate smaller and retinotopically largely non-overlapping parts of the visual field than a checkerboard. Further, V1 was activated more strongly by auditory letters than by noise bursts, which may reflect that auditory letters possess learned audiovisual associations. Both of these factors contributed to that in V1, auditory letters caused stronger activations than visual letters (i.e., cross-sensory activations were stronger than sensory-specific responses), which was not the case for the simpler stimuli. In A1, the responses to auditory letters and noise bursts were quite similar in amplitude. Further, in A1 the visual letters evoked weak BOLD activations only in the right hemisphere; for checkerboards similar weak activations were observed bilaterally.

## 4 Discussion

Early cross-sensory activations and audiovisual interactions were found in both A1 and V1. In A1 the delay from sensory-specific to cross-sensory activity was 40 ms, whereas in V1 the delay was only 14 ms (see also (Brang et al., 2015) intracranial data showing about simultaneous activation of human V1 by auditory and visual stimuli). This asymmetrical timing pattern, where sensory-specific activations start earlier in A1 (25 ms) than in V1 (48 ms) cortex, is consistent with that the cross-sensory activations originate in the sensory cortex of the opposite stimulus modality, with about 17–37 ms conduction delay between A1 and V1. Audiovisual interactions were observed only after both sensory-specific and cross-sensory inputs converged on the sensory cortex.

The present results expand our earlier results obtained using simpler stimuli (Raij et al., 2010; Ahveninen et al., 2024). Despite clearly different stimuli, the sensory-specific and cross-sensory onset latencies were quite consistent between letters and simpler stimuli. The cross-modal conduction delays were also similar. It remains plausible that the earliest cross-sensory activations may utilize the A1 → V1 and V1 → V2 → A1 pathways. However, other viable options remain, including other cortico-cortical connections between the auditory and visual cortices, connections through subcortical relays, or through an association cortical area such as STP/STS (Foxe and Simpson, 2002; Schroeder and Foxe, 2002; Schroeder et al., 2003; Liang et al., 2013; Henschke et al., 2015; Lohse et al., 2021). Perhaps in support of the last option, in the present data, left posterior STS was strongly activated at the same time as the cross-sensory auditory cortex activation occurred. However, in the right hemisphere only weak STS activity was observed. This left STS hemispheric lateralization is reverse to what was observed for simpler stimuli; it may be that different stimulus materials utilize partially different pathways, with language stimuli lateralized to the left. Further studies are needed to characterize the timing of network nodes supporting multisensory processing outside the primary sensory areas.

In accord with our previous results (Raij et al., 2010), audiovisual interactions started only after the sensory-specific and cross-sensory inputs converged at the sensory cortex (at 80 ms in the auditory cortex and 50 ms in the visual cortex, i.e., in the auditory cortex 40 ms and in the visual cortex 77 ms after convergence). However, these interactions started later for letters (present study) than for simpler stimuli (Raij et al., 2010). Whether this difference reflects physical stimulus properties (e.g., the amplitude envelopes of spoken letter names were more varied than noise bursts) or that previously learned audiovisual associations prolong the onset of interactions warrants further investigation. Yet, prior electroencephalography (EEG) evidence shows that audiovisual interactions in humans can occur earlier, starting already at about 40 ms, over posterior areas (Giard and Peronnet, 1999; Molholm et al., 2002; Teder-Sälejärvi et al., 2002; Molholm et al., 2004; Cappe et al., 2010). A possible explanation is that the earliest interactions in EEG may be generated in subcortical structures (Raij et al., 2010); EEG is more sensitive to signals from deep structures than MEG (Goldenholz et al., 2009).

The MVPA results mainly agreed with the evoked response onset latencies in terms of relative timing differences between A1 and V1 and the order of processing stages. However, it is worth noting that these MVPA latencies refer to the centroid of a sliding time window, which was utilized to classify different sensory conditions based on a dynamic pattern of brain activity. Thus, while MVPA decoding provided strong evidence of cross-modal influences in auditory and visual cortices, this analysis technique did not provide direct information on the actual neurophysiological onset latencies. Evoked response onset latencies remain better suited for this purpose.

The fMRI results largely agreed with the MEG localization results, but some discrepancies were observed. In V1, fMRI detected strong cross-sensory activations, which was in accord with MEG results. Moreover, in V1 the cross-sensory activations were stronger than the sensory-specific activations, probably reflecting that the visual letters only activated a small part of the visual field [compare these to the cross-sensory activations to checkerboards in Raij et al., 2010]. However, in A1, where MEG showed strong cross-sensory activations, fMRI showed only weak right hemisphere (for letters) or bilateral (for checkerboards) cross-sensory responses. Summarizing the fMRI findings from these two studies, it seems likely that A1 may be activated by visual stimuli. This is in agreement with an fMRI study reporting more robust A1 activations for checkerboards (Martuzzi et al., 2007), another fMRI study reporting that responses to auditory letters are modulated by simultaneously presented visual letters (van Atteveldt et al., 2004), and with our MEG findings. One explanation why the fMRI cross-sensory A1 activations in our two studies are weak may be that the acoustical scanner noise, accentuated by our rapid scanning parameters (short TR), dampened auditory cortex responses due to neuronal adaptation (in contrast, the MEG scanner is silent). Future fMRI studies using acoustically more quiet continuous EPI sequences or sparse sampling may offer further insight (Hall et al., 1999; Hennel et al., 1999; Yang et al., 2000; Schwarzbauer et al., 2006; Gaab et al., 2007a,b; Schmitter et al., 2008).

Limitations of the study include a relatively small sample size, which was dictated by the use of multiple stimulus types and ISIs, recording both MEG and fMRI data, and the resulting long recording sessions. Future studies with more subjects are needed to test if some of the differences that were non-significant in the current data could emerge as significant.

Functional roles of early cross-sensory activations are still incompletely understood. Audiovisual interactions for letters are clearly stronger after 300 ms than at these early latencies, and differentiate between matching and non-matching letter pairs even later, after 400 ms; moreover, such activations are maximal in higher-order association areas such as STS (Raij et al., 2000). Plausibly, the early cross-sensory influences in low-order sensory areas may have a role for situations where synchrony requirements may be tight, and/or could facilitate later processing stages and reaction times by speeding up the exchange of signals between brain areas and enhancing top-down processing (Ullman, 1996; Bar et al., 2006; Raij et al., 2008; Sperdin et al., 2009). They may also be behaviorally particularly relevant in, e.g., situations where stimuli in one or more modalities are noisy (Jääskeläinen et al., 2011; Schepers et al., 2015; Bizley et al., 2016; Ahveninen et al., 2024). Overall, the fact that the onset latency differences between simpler stimuli and letters were small agrees with the view that the earliest cross-modal activations in sensory cortices reflect relatively automatic bottom-up processes (van Atteveldt et al., 2014; De Meo et al., 2015).

The observed cross-sensory onset latencies are, to our knowledge, the fastest reported for letter stimuli, and both these and sensory-specific latencies are consistent with those previously reported for simpler stimuli (Raij et al., 2010; Ahveninen et al., 2024). These findings contribute to understanding the timing and potential anatomical pathways of early cross-sensory activations and interactions in sensory cortices in language processing. Further, the present quantification of millisecond-level cross-sensory conduction delays may enable future studies that manipulate effective connectivity between the stimulated brain areas (Hernandez-Pavon et al., 2022).

## Data Availability

The raw data supporting the conclusions of this article will be made available by the authors, without undue reservation.

## Footnotes

^1^http://www.cosmomvpa.org/
