# Supplementary material for: Onset timing of letter processing in auditory and visual sensory cortices
Source: Front Integr Neurosci. 2024 Nov 14;18:1427149. doi: 10.3389/fnint.2024.1427149 (PMC11602476; doi:10.3389/fnint.2024.1427149)
Supplement: Supplementary file 1 [file Table_1.docx]

Supplementary Material

**Onset timing of letter processing in auditory and visual sensory cortices by Raij *et al.***

### Supplementary Materials and Methods

The total number of presented stimuli was 375 for each individual subject and stimulus modality (A/V/AV). Some epochs were discarded from averaging based on that they contained artifacts (signal amplitudes exceeding 150 µV or 3000 fT/cm at any EEG/EOG or MEG channel, respectively). Data from one subject was discarded due to noise (same subject was discarded in (Raij et al., 2010)). Additionally, in two subjects, one ISI run (out of the total of 3) was rejected entirely due to excessive eye blink artifacts. The number of accepted epochs for computation of the evoked response of each subject (mean ± SD) was 277 ± 84 (range 149 – 357, median 308) for auditory stimuli, 279 ± 81 (range 166 – 356, median 318) for visual stimuli, and 268 ± 81 (range 159 – 357, median 293) for audiovisual stimuli. The total number of epochs in the grand average signal was 1938 for auditory, 1954 for visual, and 1879 for audiovisual stimuli.

The MEG sensor-space results were extracted as follows: First, the evoked responses were calculated for each of the MEG gradiometers separately. The MEG instrument contains 204 gradiometers at 102 locations. Each location contains a gradiometer pair *x* and *y* with an in-plane rotation of 90 degrees between them. These record the two orthogonal components of the magnetic field gradient *b_x_* and *b_y_* at each location. To calculate the response amplitude at each location, the information from the two sensors x and y needs to be combined to avoid potential bias resulting from different subjects having neuronal generators with different orientations. Following the Pythagorean Theorem, the amplitudes were computed as $\surd(b_{x^{2}} + b_{y^{2}})$ separately at each of the 102 locations. Finally, for the grand average responses in Figure 1, three sensor locations (over left A1, right A1, and midline V1) showing the maximal responses for A and V unimodal stimuli were selected and averaged across subjects.

The grand average onset values in source space were extracted with bootstrapping (B=5000) to estimate the mean, variance, and median. Specifically, evoked responses to each stimulus category were first averaged across the left and right hemisphere, after which the population formed by each ISI condition (N=3 except for two subjects N=2) and subject (N=7) formed the population (N=19) for which bootstrapping was performed. Using bootstrapping for this was particularly useful for the interaction response $\left[ AV - \left( A +V \right) \right]$ due to its stronger noise than in the constituent responses A/V/AV. **Table 3** shows the results of this analysis for all stimulus categories. Further, the onset latencies for A/V/AV stimuli (but not the interaction response, due to SNR considerations) were estimated directly from the individual subjects’ evoked responses (**Table 4**). For interpretation see Discussion.

Due to the SNR considerations described above, testing the differences between AV interactions for (i) letters and (ii) simpler stimuli also relied on bootstrapped data of the source-space AV interaction response time courses. Separately for A1 and V1, the onset latencies were extracted from each iteration of the bootstrap (N = 5000) to create two distributions, one for letters and the other for simpler stimuli. Then, the Wilcoxon Sign Rank Test was used to assess if the interaction response onsets significantly differed between letters and simpler stimuli.

**Supplementary References**

Raij, T., Ahveninen, J., Lin, F., Witzel, T., Jääskeläinen, I., Letham, B., Israeli, E., Sahyoun, C., Vasios, C., Stufflebeam, S., Hämäläinen, M., and Belliveau, J. (2010). Onset timing of cross-sensory activations and multisensory interactions in auditory and visual sensory cortices. *Eur J Neurosci* 31**,** 1772-1782.
